# Exclusive Breastfeeding Practice and Associated Factors among Mothers Attending Private Pediatric and Child Clinics, Addis Ababa, Ethiopia: A Cross-Sectional Study

**DOI:** 10.1155/2017/8546192

**Published:** 2017-12-03

**Authors:** Laykewold Elyas, Amha Mekasha, Amha Admasie, Etagegnehu Assefa

**Affiliations:** ^1^Department of Pediatrics and Child Health, College of Medicine and Health Sciences, Addis Ababa University, Addis Ababa, Ethiopia; ^2^School of Public Health, College of Health Sciences and Medicine, Wolaita Sodo University, Wolaita Sodo, Ethiopia; ^3^Department of Chemistry, College of Natural and Computational Science, Wolaita Sodo University, Wolaita Sodo, Ethiopia

## Abstract

**Background:**

The prevalence of exclusive breastfeeding (EBF) is globally low (35%) in sub-Saharan Africa, whereas it is 58% in Ethiopia. Exclusive breastfeeding has the potential to prevent 11.6% of under-five deaths in developing countries. Therefore, the main objective of this study was to assess the exclusive breastfeeding practice and associated factors on mothers attending private pediatric and child clinics in Addis Ababa, Ethiopia.

**Methods:**

An institutional-based cross-sectional study design was used. A total of 380 samples were obtained. Data were collected using a self-administered questionnaire. Data was entered and analyzed using SPSS version 16. Descriptive statistics and logistic regression analysis were used.

**Results:**

From 380 mothers, only 44.2% of the mothers practiced EBF. Two hundred (52.6%) mothers started breastfeeding within 1 hour of delivery; 161 (42.4%) of the mothers gave extra food before six months, and 244 (64.2%) believed that exclusive breastfeeding was sufficient. Moreover, 288 (75.8%) mothers breastfed their children eight or more times per day. Spontaneous vaginal delivery was a significant factor to practice EBF (AOR: 1.86, 95% CI: 1.19–2.89).

**Conclusion:**

EBF practice in this study was low. Spontaneous vaginal delivery was a significant factor for EBF; hence, it is very crucial to promote EBF.

## 1. Background

Exclusive breastfeeding (EBF) has been defined by the WHO as the situation where “the infant has received only breast milk from his/her mother or a wet nurse, or expressed breast milk and no other liquids, or solids, with the exception of drops or syrups consisting of vitamins, minerals, supplements, or medicines” [1]. Breastfeeding is a natural food that serves as a complete source of infant nutrition for the first six months of life. It contains all the necessary nutrients provided in a bioavailable and easily digestible form, protecting both mothers and children against illnesses and diseases with immunological properties [2]. Breast milk contains essential fatty acids needed for the infant's growing brain, eyes, and blood vessels and these are not available in other types of milk. Breastfeeding on demand at day and night at least 8 times in 24 hours will provide more milk as suckling stimulates milk production [3]. Infants that are exclusively breastfed have a lower chance of becoming ill or dying from diarrhea and infections and are less likely to acquire pneumonia, meningitis, and ear infections than those that were not exclusively breastfed [4]; this is protective against single and recurrent incidences of otitis media in particular and the risk of hospitalization for lower respiratory tract infections during the first year of life. In addition, breastfeeding also benefits the society by reducing healthcare costs, parental employee absenteeism, and the associated loss of family income [5, 6].

Tampah-Naah and Kumi-Kyereme reported that human breast milk is the healthiest form of milk for human babies with few exceptions, such as when the mother is taking certain drugs or is infected with tuberculosis or HIV [7]. Kong and Lee posited that breast milk is produced under the influence of prolactin and oxytocin; women produce milk after child birth to feed the baby. The initial milk produced is often referred to as colostrum, which is rich in immunoglobulins that coat the gastrointestinal tract. This helps protect the newborn until his/her own immune system starts properly functioning and creates a mild laxative effect, expelling meconium and helping to prevent the buildup of bilirubin (a contributory factor in jaundice) [8]. Breastfeeding increases oxytocin levels which also contribute to maternal-child bonding, lowering the risk of developing uterine cancer, osteoporosis, type two diabetes, and breast cancer in women [9].

The World Health Organization (WHO) and UNICEF also recommend that all mothers should breastfeed their children exclusively for the first six months of life [10, 11]. It is recommended that breastfeeding should begin within one hour after birth. Early initiation of breastfeeding should be promoted and prelacteal feeds should be discouraged. Because of its high levels of vitamin A, antibodies, and other protective factors, the colostrum is often considered the baby's first immunization [3]. Simple, valid, and reliable indicators are essential to track the progress and guide investment to improve nutrition and health during the first two years of life. Of the indicators, exclusive breastfeeding ranks first, being estimated to have the potential to prevent 13% of all deaths [12]. Indeed, of the 6.9 million under-five children that were reported to be dead globally in 2011, an estimated 1 million lives could have been saved by simple and accessible practices such as EBF [12]. Consequently, the WHO and UNICEF (1990) have recommended EBF for six months, followed by introduction of complementary foods and continued breastfeeding for 24 months or more [10]. Despite this initiative, breastfeeding has not reached the desired marks. One major reason often posed for mothers not practicing exclusive breastfeeding is the inability of the nursing mother to lactate sufficiently [13].

Current analyses on global incidence across 140 countries reported an increase in breastfeeding practice in the developing world from 33% in 1995 to 39% in 2010 among infants aged 0–5 months and also an improvement from 35% in 1995 to 47% in 2010 along with countries in Eastern and Southern Africa [14].

According to the report of Leung and Suave, maternal age was significantly associated with initial BF and showed clear dose-response gradients with older ages [15]. Another study conducted in rural China reported that mothers with education level above senior middle school were less likely to exclusively breastfeed their infants [16].

A study conducted on 10,519 mothers in the US [17] indicated that women with a higher family income level were more likely to exclusively breastfeed their infants than their lower income counterparts. Inversely, studies from Saudi Arabia, Peru, and the Philippines [18] all found that higher family income was associated with a reduced probability of initiation and duration of breastfeeding. In a study on infant feeding practice among nursing personnel in Australia, many women identified employment as a barrier to exclusive breastfeeding practice, and returning to work was one of the reasons why women ceased breastfeeding, with 60 percent of women intending to breastfeed when they return to work, but only 40 percent did so [19].

Nutrition and medical journals have added to the body of research that states simply and emphatically that breast milk's health and developmental benefits far surpass any artificial infant formula [20]. A study in Peru shows that infants under six months of age who were not breastfed had a fourfold greater risk of developing acute respiratory infections compared with exclusively breastfed babies [9].

Low practice of EBF in Ethiopia is attributed to various maternal and child factors, such as having an infant aged 2-3 months, giving birth in a health facility, being a housewife in occupation, receiving counseling/advice on infant and colostrum's feeding, which were contributing factors to practice EBF [21]. Age of the mother and access to postnatal care were also encouraging to practice EBF [22]; mothers who received breastfeeding counseling during pregnancy and being supported by the husband were also motivational factors to practice EBF [23, 24]. Better maternal education, marital status, good wealth index, and lower age of the child [25] were more likely to practice exclusive breastfeeding than their counterparts.

According to a study on female medical doctors in Nigeria, only 11% of mothers practiced exclusive breastfeeding for six months [2]. In many countries including Ethiopia, EBF practice is lower than the international recommendation [26]. Many studies in Ethiopia indicated the different prevalence of EBF in different areas of the country: EDHS 2005, 49% [27]; EDHS 2011, 52%, with a mean duration of 4.2 months [28]; Debre Tabor, Amhara Region, Ethiopia, 70.8% [21]; Enderta, Tigray Region, Ethiopia, 70.2% [22]; and Motta (East Gojjam), Amhara Region, Ethiopia, 50.1% [23]. EBF in the last 24 hours preceding the survey in Goba town, Oromia Region, Ethiopia, was 71.3% [24]. A study done in Gondar, Ethiopia, among female nurses and midwives revealed that 35.9% practiced EBF for six months, and the mean duration of EBF practice was 4.1 ± 1.7 months [29].

Based on a few lines of evidence, EBF practice in Ethiopia is poor, which is contrary to the WHO recommendations. Most researches on EBF were conducted in regional states of Ethiopia, but none of these studies included women who attended private clinics. Therefore, the purpose of this research was to assess the prevalence and associated factors of EBF practice in private pediatric and child clinics, Addis Ababa, Ethiopia.

## 2. Method

### 2.1. Study Area and Period

The study area of this research is Addis Ababa which is the capital of Ethiopia. The study was conducted from 1 March to 31 May 2016. There are around 42 hospitals and 21 private clinics in Addis Ababa city. Yeka subcity is one among 10 subcities of Addis Ababa city. The residents of Addis Ababa especially living in Yeka subcity and the surrounding area were the geographic targets covered in the study. Since Yeka subcity and the nearby residents are the frequent customers for the clinic because of the geographical proximity, those who are the users of this clinic service are the direct study population from whom samples have been drawn.

### 2.2. Study Design and Population

An institutional-based cross-sectional descriptive study design was used to collect data from mothers who were attending private pediatric and child clinics. The source population were all mothers who were residents of Addis Ababa, who were private clinic users for antenatal care services. The study population were all mothers who were breastfeeding and visited the clinic for different antenatal care services. But mothers who had repeated visits during the study period were excluded from the study.

### 2.3. Sample Size Determination

The sample size was estimated using a single population proportion formula assuming an expected prevalence for exclusive breastfeeding in Ethiopian demographic and health survey study to be 52% [28] with a 95% confidence level, at a 5% margin of error, adding a nonresponse rate of 10%. The calculated sample size using the above assumptions became 383. By considering a 10% nonresponse rate, the overall minimum sample size was 421.

### 2.4. Sampling Techniques and Approach

Not all mothers who were coming to the clinic have been included as part of the study because of logistic feasibility. On average, 600 breastfeeding mothers per month were expected to visit the clinic for antenatal care services. Taking a total of 3 months, 1800 mothers were estimated to visit the clinic. Therefore, a sample was taken from this population within the 3 months' duration. Therefore, data was collected from every five visiting breastfeeding mothers. Based on the exclusion criteria, mothers who came to the clinic more than once during the study period were excluded to avoid repetition for data collection.

### 2.5. Data Collection Techniques

The data was collected using a self-administered questionnaire to the mothers. For those mothers who are able to read and write, the questionnaire was given to them and they filled it out by themselves freely, but for those mothers who faced difficulties in understanding the questions, they were assisted by the data collectors. The questionnaire was read for them and their response was recorded. The questionnaire was adopted from different literatures so as to suit the local context. The questionnaire was designed with both close-ended and open-ended types of questions.

### 2.6. Data Analysis Technique

The collected data was cleaned, coded, and entered into EpiData software version 3.1 and then exported to the Statistical Package for the Social Sciences (SPSS) version 16 for Windows for further analysis. Descriptive statistics such as frequencies, proportions, and measures of central tendency and measures of variation were used to describe the distributions of variables. The logistic regression model was used to assess the relation between dependent and explanatory variables. Bivariate analysis was done to examine the associations of single independent variable with exclusive breastfeeding practice.

Independent variables with *P* value ≤ 0.25 during bivariate analysis were entered into the multivariate analysis model, for the exclusive breastfeeding practice. Association between dependent and independent variables was assessed using AOR with 95% CI. Statistical association was declared significant when the *P* value was less than 0.05. Multicollinearity was checked using a cutoff point based on variance inflation factor (VIF) < 10 and tolerance test > 0.1. Hosmer and Lemeshew's goodness-of-fit test was used to assess the fitness of the model.

### 2.7. Variables

#### 2.7.1. Dependent Variables

The dependent variable was exclusive breastfeeding practice of mothers who were attending a healthcare service in a private pediatrics and child health clinic in Addis Ababa, Ethiopia.

#### 2.7.2. Independent Variables

Socioeconomic/demographic factors such as child's age and maternal socioeconomic characteristics, knowledge and attitude towards EBF of the mother, knowledge about the importance of EBF, knowledge about extra feed for the children including the time to start, and factors affecting the prevalence of EBF are the main variables addressed in this research.

### 2.8. Operational Definition

#### 2.8.1. Exclusive Breastfeeding

The infant has received only breast milk from his/her mother, or expressed breast milk and no other liquids, or solids, with the exception of drops or syrups consisting of vitamins, minerals supplements, or medicines.

### 2.9. Ethical Issues

The ethical approval for this study was obtained from the Department of Pediatrics and Child Health, School of Medicine, Addis Ababa University. Mothers have been asked for their consent verbally to confirm their willingness to participate in the study. Prior to enrolling any of the eligible study participants, the purpose, the benefits, and the confidential nature of the study were described and discussed with each participant. Confidentiality was ensured by omitting the names of the respondents from the questionnaire. Only those that consented and proved their willingness to take part in the study were enrolled in the study. Child care and nutritional advice was given to mothers by the data collectors during the data collection period.

## 3. Result

A total of 421 questionnaires were distributed to mothers attending private pediatric clinics. However, 380 questionnaires were found complete for analysis. The remaining 41 of the questionnaires were not fully answered correctly by the respondents.

### 3.1. Sociodemographic Information about the Respondents

Regarding the profile of the mothers' age distribution, 90 (23.7%) of them were in the age group of 15–25 years, whereas 259 (68.2%) of them were in the age group of 26–35 years. The remaining 31 (8.2%) were 36–45 years of age and above. Majority of the mothers (349, 91.8%) gave birth when they were below the age of 36. Regarding the religion of mothers, 293 (77.1%), 55 (14.5%), and 26 (6.8%) were Orthodox Christians, Protestants, and Muslims, respectively.

Regarding mothers' education status, it was also found that 14 (3.7%) were able to read and write, 108 (28.4%) completed either primary or secondary school, 131 (34.5%) had a diploma level of education, and 127 (33.4%) had a degree and were masters graduates.

In terms of the means of income for the mothers, 210 (55.3%) were living with the income of their husbands, whereas the rest (170, 44.7%) had their own means of income, which means they were either employed or had their personal business. The amount of monthly income ranges from a minimum of 7000.00 ETB to more than 15000.00 ETB. 161 (42.4%) of the mothers had a monthly income above 15000.00 ETB, whereas 145 (38.2%) had a monthly income of 11000.00–15000.00 ETB (Table 1).

### 3.2. Child Profile

The age distributions of female and male children were more or less equal, 195 (51.3%) females and 185 (48.7%) males, but the females' proportion was a bit more than the males'. Regarding the age of the child, 262 (68.9%) of the children were aged between 7 and 24 months, whereas 64 (16.8%) of the children were 3 months of age or less. Almost all mothers (377, 99.2%) gave birth in health institutions like hospitals, health centers, and specialized clinics, while the rest of them gave birth at home. As it is a big city with a relatively civilized community, it is not tolerable for a mother to give birth at home in a normal circumstance (Table 2).

### 3.3. Exclusive Breastfeeding, Complimentary Feeding, and Prenatal Status

Among mothers performing the exclusive breastfeeding practice, 168 (44.2%) had EBF for 6 months or more, while 212 (55.8%) provided EBF before 6 months of age. Two hundred (52.6%) mothers started to breastfeed within 1 hour of delivery; the rest (114, 30%) started breastfeeding after 1 hour of delivery. Hence, children from these mothers (114, 30%) are assumed to have a lack of important elements obtained from their mothers' breast milk because of the delay in starting breastfeeding. Vitamin A, antibodies, and other protective factors are highly important for the children, but the mothers do not have this awareness. Sixty-six (17.4%) mothers even did not remember when they started breastfeeding. Some mothers indicated that the child was not willing to start breastfeeding right after delivery.

Concerning the mode of delivery of the mothers, 193 (50.8%) delivered by SVD and the rest (187, 49.2%) gave birth by C/S.

Regarding extra foods, 161 (42.4%) of the respondents gave extra food for their child before six months. Two hundred forty-four (64.2%) mothers in this study knew that EBF was sufficient for their child, while the rest (136, 35.8%) believed that EBF was not sufficient for their child. Moreover, 288 (75.8%) mothers breastfed their child 8 or more times per day (Table 3).

### 3.4. Bivariate and Multivariate Logistic Regression

Numerous predictors of exclusive breastfeeding practice before six months in under-five children were assessed using logistic regression model. Each potential predictor variable was analyzed using binary logistic regression, and hence the age of the child and mother, religion of the mother, means of income of the mother, mode of delivery, experience of fluid giving before six months, and thinking that EBF is sufficient for six months were shown to have a significant association with the dependent variable (*P* < 0.25). Furthermore, significantly associated variables were entered to the multivariate logistic regression model using Enter method of regression. As a result of multivariate logistic regression, only the mode of delivery was shown to have a significant association with the exclusive breastfeeding practice at *P* < 0.05 (Table 4).

## 4. Discussion

Based on the study, the prevalence of exclusive breastfeeding practice was 168 (44.2%); this was lower than the value by Ethiopian Demographic and Health Surveys 2005, which was 49% [27], EDHS 2011 which was 52% with a median duration of 2.3 months and mean duration of 4.2 months [28], EDHS 2016 which was 58% [30], and Motta (East Gojjam), Amhara Region, Ethiopia, which was 50.1% [23]. But this value was by far lower than those in studies in Enderta, Tigray Region, Ethiopia, 70.2% [22]; Debre Tabor town, Amhara Region, Ethiopia, 70.8% [21]; and EBF in the last 24 hours preceding the survey in Goba town, Oromia Region, Ethiopia, 71.3% [24]. The difference could be due to the difference in health information and awareness. The World Health Organization (WHO) and the United Nations Children's Fund (UNICEF) recommend that all mothers should breastfeed their children exclusively for the first 6 months and thereafter they should continue to breastfeed for as long as the mother and child wish, and both appropriate and sufficient weaning food should be added after six months of life [16]; the WHO Global and National Infant and Young Child Feeding guidelines recommend that all newborns should be exclusively breastfed for the first six months [16].

52.6% of the mothers started breastfeeding their newborn child within 1 hour. The rest (30%) started breastfeeding after 1 hour. Even 17.4% of the study population did not remember when they started breastfeeding. The WHO and UNICEF recommend that all mothers should breastfeed their children exclusively for the first six months of life. It is also recommended that breastfeeding should begin within one hour after birth. Early initiation of breastfeeding should be promoted and prelacteal feeds should be discouraged. Because of its high levels of vitamin A, antibodies, and other protective factors, the colostrum is often considered as the baby's first immunization [16]. This study has insignificant differences with other studies regarding the initiation of breastfeeding within one hour: Debre Tabor town, Amhara Region, Ethiopia, 78.6% [21], Enderta (woreda), Tigray Region, Ethiopia, 65% [22]. Hence, children of these mothers (30%) who initiated breastfeeding after 1 hour missed the important elements to be obtained from the mothers' milk because of the delay in starting breastfeeding. Of course, the cause of the delay in initiation of breastfeeding was not only the mothers' lack of awareness but also the fact that some mothers believed that the child was not willing to start breastfeeding right after delivery.

The median duration of EBF in the study area was found to be 0.8 months, which is comparable to EDHS 2011 where the median duration for Afar Region of Ethiopia was 0.6 months. This result is very low compared to many regions with a median ranging from 1 month (Addis Ababa) to 4.6 months (Amhara Region) [28], also lower as compared to Addis Ababa, 2.9 months of median duration [30]. This result is an alarm necessitating a significant effort from the responsible bodies on the problem.

Regarding the impact of knowledge of the mothers on whether they should feed their child extra fluid before six months, although their knowledge is generally good about the importance of EBF, they did not practice it to the required level. As discussed before, knowing the importance is not sufficient. Since other factors like employment and parity affect the prevalence of EBF, mothers do not follow the recommended EBF guidelines recommended by the WHO and UNICEF. Employed mothers have a lower opportunity to stay at home, compromising exclusive breastfeeding. Mothers also may have to leave their babies to search for a job. It was found that only 43% of employed mothers breastfed their child for six months, whereas the unemployed figure is 56%, which is 13% more than the employed figure. The workload that employed mothers have affects the prevalence of EBF.

Unfortunately, most factors which are known to be a contributing factor in other studies were not significantly associated with EBF in this study. But only delivery type has a significant influence on the EBF; a mother who delivered by SVD is 1.86 times more likely to practice exclusive breastfeeding than the mother who had a C/S delivery (AOR: 1.86, 95% CI: 1.19–2.89). This study was consistent with the study done by Tewabe et al. (mothers that delivered by SVD were 2.06 times more likely to practice EBF than those that delivered by C/S [23]) and Setegn et al. (mothers that delivered by SVD were 1.7 times more likely to practice EBF than those that delivered by C/S [24]). But this study was contradicting with the study where a mother who delivered by SVD was 0.48 times less likely to practice exclusive breastfeeding than a mother who had a C/S delivery [21]. Delivery type (i.e., whether spontaneous or by Cesarean section) has been found to influence both EBF initiation and duration. While one group of researchers have reported positive associations between delivery type (vaginal delivery) and EBF, others have found an inverse association. In Western Australia, it was found that mothers who had a vaginal delivery were about twice as likely to exclusively breastfeed at hospital discharge compared to mothers who had Cesarean delivery [19]. The reason could be that the mother who had a C/S delivery was less comfortable and felt fatigued after the C/S procedure to give her breast to her newborn.

## 5. Conclusion

Exclusive breastfeeding (EBF) practice in this study was lower than in most of the studies. Mode of delivery (spontaneous vaginal delivery) was indicated as the determinant factor for exclusive breastfeeding practice of mothers.

## Figures and Tables

**Table 1:** Sociodemographic characteristics of mothers attending private pediatric and child clinics, Addis Ababa, Ethiopia, March–May 2016.

Variable (*n* = 380)	Respondents' response	Frequency	Percent
Age of mother	15–25 years	90	23.7
	26–35 years	259	68.2
	36–45 years and above	31	8.2
Religion of mother	Orthodox	293	77.1
	Muslim	26	6.8
	Protestant	55	14.5
	Other	6	1.6
Educational status of mother	Literate	14	3.7
	Primary to secondary school	108	28.4
	Diploma	131	34.5
	First degree	106	27.9
	Masters and above	21	5.5
Parental employment status	Employed	218	57.4
	Unemployed	162	42.6
Marital status	Married	362	95.3
	Unmarried	13	3.4
	Divorced/widowed	5	1.3
Means of household income	I have my own income	170	44.7
	Only my husband's income	210	55.3
Amount of monthly income	7001–11000 ETB^*∗*^	74	19.5
	11001–15000 ETB	145	38.2
	>15000 ETB	161	42.4

^*∗*^ETB: Ethiopian Birr.

**Table 2:** Child profile of mothers attending private pediatric clinics, Addis Ababa, Ethiopia, March–May 2016.

Variable with respondents' response (*n* = 380)	Frequency	Percent
Sex of the child		
Female	195	51.3
Male	185	48.7
Age of the child		
≤3 months	64	16.8
4–6 months	54	14.2
7–24 months	262	68.9
Birth place of the child		
Home	3	0.8
Health institution	377	99.2

**Table 3:** Mothers' experience of breastfeeding and giving supplementary foods practice of mothers attending private pediatric clinics, Addis Ababa, Ethiopia, March–May 2016.

Variables with respondents' response (*n* = 380)	Frequency	Percent
How long have you provided only breastfeeding?		
≥6 months	168	44.2
<6 months	212	55.8
Have you given any fluid before six months besides breastfeeding?		
Yes	161	42.4
No	219	57.6
Parental delivery system		
SVD	193	50.8
C/S	187	49.2
Parity status of mothers		
1st	203	53.4
2nd	107	28.2
3rd	49	12.9
4th	17	4.5
5th	4	1.1
When was breastfeeding started?		
Within 1 hour	200	52.6
After 1 hour	114	30.0
I do not remember	66	17.4
Have you given any fluid before six months besides breastfeeding?		
Yes	161	42.4
No	219	57.6
Do you think that EBF is sufficient for your child for the first 6 months?		
Yes	244	64.2
No	136	35.8
I think that my child will face a health problem if only EBF is given for long		
Yes	249	65.5
No	131	34.5
How many times a day do you breastfeed your child?		
6 times per day	31	8.2
7 times per day	17	4.5
8 times per day	44	11.6
>8 times per day	288	75.8
I think that my child will face a health problem if only EBF is given for long		
Yes	249	65.5
No	131	34.5
Do you think that breastfeeding for long is important for the health and strength of the child?		
Yes	375	98.7
No	5	1.3

**Table 4:** Bivariate and multivariate logistic regression of factors affecting EBF of mothers attending private pediatric clinics, Addis Ababa, Ethiopia, March–May 2016.

	EBF practice		COR (95% CI)	AOR (95% CI)
	Yes	No		
Age of child				
≤3 months	26	38	0.85 (0.49–1.48)	0.84 (0.47–1.49)
4 months	14	10	1.74 (0.74–4.05)	1.76 (0.72–4.30)
5 months	2	11	0.23 (0.05–1.04)	0.17 (0.03–0.80)^*∗*^
6 months	9	8	1.39 (0.52–3.73)	1.38 (0.49–3.85)
7–24 months	117	145	1.00	1.00
Age of mother				
15–25 years	34	56	1.10 (0.47–2.58)	0.79 (0.32–1.94)
26–35 years	123	136	1.64 (0.76–3.57)	1.60 (0.71–3.59)
36+ years	11	20	1.00	1.00
Religion of mother				
Orthodox	121	172	0.70 (0.14–3.54)	0.70 (0.13–3.69)
Muslim	18	8	2.25 (0.37–13.67)	1.97 (0.30–12.83)
Protestant	26	29	0.90 (0.17–4.84)	1.08 (0.19–6.07)
Other	3	3	1.00	1.00
Do mothers have their own means of income?				
My own income	69	101	0.77 (0.51–1.15)	0.74 (0.48–1.16)
With my husband	99	111	1.00	1.00
Mode of delivery				
SVD	97	96	1.65 (1.10–2.48)	1.86 (1.19–2.89)^*∗*^
C/S	71	116	1.00	1.00
Have you given any fluid before six months besides breastfeeding?				
Yes	64	97	0.73 (0.48–1.10)	0.77 (0.50–1.20)
No	104	115	1.00	1.00
Do you think that EBF is sufficient for your child for the first 6 months?				
Yes	115	129	0.72 (0.47–1.10)	0.77 (0.49–1.21)
No	53	83	1.00	1.00

^*∗*^Significance at *P* < 0.05.

## Additional Points

*Recommendation*. It is so crucial that EBF has to be promoted a lot. For this realization, the Federal Ministry of Health of Ethiopia, local and international NGOs working on health areas, and health institutions should play their role to enhance exclusive breastfeeding practice of mothers with training during antenatal and postnatal visits for women. Health personnel who are working in the clinics should advise mothers to have a spontaneous vaginal delivery and should not insist on performing C/S delivery unless and otherwise medically justified.* Limitations of the Study*. This study was conducted in a single private pediatric and child clinic, which could lead to less plausible generalization and can be considered as the main limitation of the study. Besides, recall bias and social desirability bias were critical manifestations in self-reported responses.
